# Adenosine infusion increases plasma levels of VEGF in humans

**DOI:** 10.1186/1472-6793-5-10

**Published:** 2005-06-20

**Authors:** Thomas H Adair, Reid Cotten, Jian-Wei Gu, Janelle S Pryor, Kenneth R Bennett, Michael R McMullan, Preston McDonnell, Jean-Pierre Montani

**Affiliations:** 1 Angiogenesis Research LaboratoriesCenter for Excellence in Cardiovascular-Renal Research; 2 Department of Physiology & Biophysics University of Mississippi Medical Center Jackson, MS 39216, USA; 3 Department of Medicine University of Mississippi Medical Center Jackson, MS 39216, USA; 4 Institute of PhysiologyUniversity of Fribourg, 1700 Fribourg, Switzerland

## Abstract

**Background:**

Many *in vitro *studies have shown that adenosine (Ado) can induce vascular endothelial growth factor (VEGF) mRNA and protein expression and stimulate endothelial proliferation. In the present study, we seek to determine whether Ado can increase circulating levels of VEGF protein in the intact human.

**Methods:**

Five outpatients 49.3 ± 6.7 years of age and weighing 88.2 ± 8.5 kg were selected. They were given a 6 min intravenous infusion of Ado (0.14 mg kg^-1 ^min^-1^) in conjunction with sestamibi myocardial perfusion scans. Mean blood pressure (MBP, calculated from systolic and diastolic values) and heart rate (HR) were determined before Ado infusion and every 2 min for the next 10 min. Plasma VEGF concentrations (ELISA) were determined immediately before Ado infusion and 1 h, 2 h, and 8 h after the infusion.

**Results:**

Plasma VEGF concentration averaged 20.3 ± 2.0 pg ml^-1 ^prior to Ado infusion, and increased to 62.7 ± 18.1 pg ml^-1 ^at 1 h post- infusion (p < 0.01). VEGF plasma concentration returned to basal levels 2 h after infusion (23.3 ± 3.4 pg ml^-1^). MBP averaged 116 ± 7 mmHg and heart rate averaged 70 ± 7 prior to Ado infusion. MBP decreased by a maximum of ~22% and HR increased by a maximum of ~17% during the infusion.

**Conclusion:**

We conclude from these preliminary findings that intravenous infusion of adenosine can increase plasma levels of VEGF in humans.

## Background

It is well-established that adenosine is an important physiological regulator for maintaining oxygen homeostasis in the heart (Berne, 1980; Ely & Berne, 1992). Adenosine formation increases when the oxygen requirements of the myocardium exceed the oxygen supply, which leads to an increase in adenosine levels in the tissues (Sparks & Bardenheuer, 1986; Duessen *et al. *1988). The adenosine released under these conditions is thought to play an important role in reestablishing a balance between oxygen supply and demand (Schrader, 1990) by virtue of its potent vasodilatory (Stepp *et al*. 1996) and anti-adrenergic effects (Neumann *et al. *1995; Dobson *et al. *1986).

Mounting evidence suggests that adenosine may also have a long-term role to increase tissue oxygenation by stimulating angiogenesis (Teuscher & Weildich, 1985; Adair *et al. *1990; Ethier *et al. *1993; Marshall, 2001; Adair, 2005). Adenosine can stimulate endothelial proliferation *in vitro *(Meininger *et al. *1988; Ethier & Dobson, 1997; Grant *et al. *1999; Gu *et al. *1999) as well as angioge nesis in a variety of *in vivo *models (Adair *et al*. 1989; Adair *et al. *1991; Adair, 2005). Although the angiogenic effects of adenosine are poorly understood, it is known that adenosine, mediated by way of adenosine A_2 _receptors can upregulate VEGF mRNA and protein expression in many different cell types (Hashimoto *et al. *1994; Takagi *et al. *1996; Grant *et al. *1999; Gu *et al. *1999; Gu *et al. *2000; Feoktistov *et al. *2002; Leibovich *et al. *2002; Feoktistov *et al. *2003; Gardener & Olah, 2003). Numerous investigators have postulated that this adenosine-mediated expression of VEGF might have a role in mediating adenosine-induced angiogenesis ; however, it is still not clear whether adenosine can induce VEGF expression in the intact organism.

In this study, we sought to determine whether adenosine can increase circulating VEGF protein in humans. The results show that a short-term intravenous infusion of adenosine caused the plasma levels of VEGF protein to increase by about threefold at one hour after the infusion

## Results

**Figure 1:**
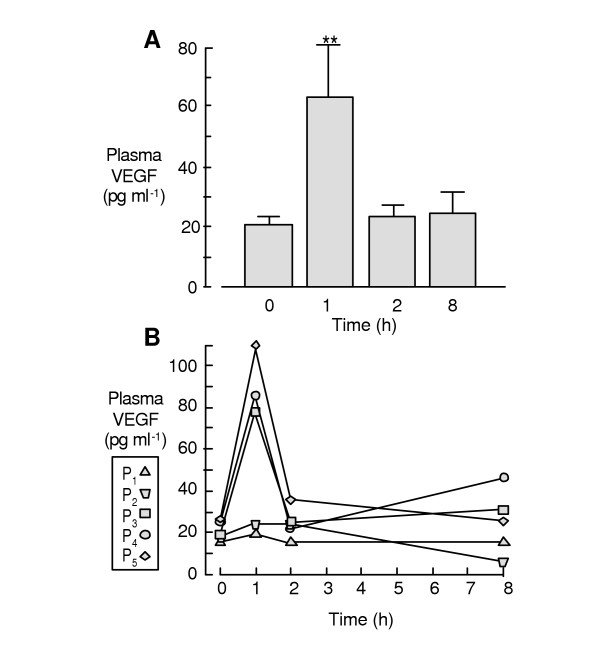
Effect of adenosine on plasma VEGF protein concentration. Adenosine (0.14 mg kg^-1 ^min^-1^) was infused intravenously for 6 minutes at 0 h. *Panel A*: Adenosine caused plasma VEGF protein concentration to increase by an average of ~3-fold at 1 h post-infusion. Plasma VEGF protein levels at 0, 1, 2, and 8 h were 20.3 ± 2.0, 62.7 ± 18.1, 23.3 ± 3.4, and 24.3 ± 7.0 pg ml^-1^, respectively. *Panel B*: Adenosine caused plasma levels of VEGF protein to increase in all 5 subjects one h after the infusion; however, the response was relatively small in two subjects. Bars are means ± S.E.M. (n = 5 for each bar). Asterisks indicate p < 0.01.

The intravenous infusion of Ado (0.14 mg kg^-1 ^min^-1 ^for 6 min) caused the plasma levels of VEGF protein to increase (P_1_–P_5_) one hour after the infusion (Figure 1a). However, the VEGF response to Ado varied greatly among the subjects ranging from a 21% increase (P_1_, 15.6 pg ml^-1 ^to 18.9 pg ml^-1^) to a 329% increase (P_5_, 25.6 pg ml^-1 ^to 110.1 pg ml^-1^), as shown in Figure 1b. Plasma VEGF protein levels averaged 20.3 ± 2.0 pg ml^-1 ^before the infusion, and increased to an average of 62.7 ± 18.1 pg ml^-1 ^(p < 0.05) one hour after the infusion. VEGF protein levels returned to control values by 2 h after the Ado infusion (23.3 ± 3.4 pg ml^-1^) and remained at control levels at 8 h post- infusion (24.3 ± 7.0 pg ml^-1^), as shown in Figure 1a.

**Figure 2:**
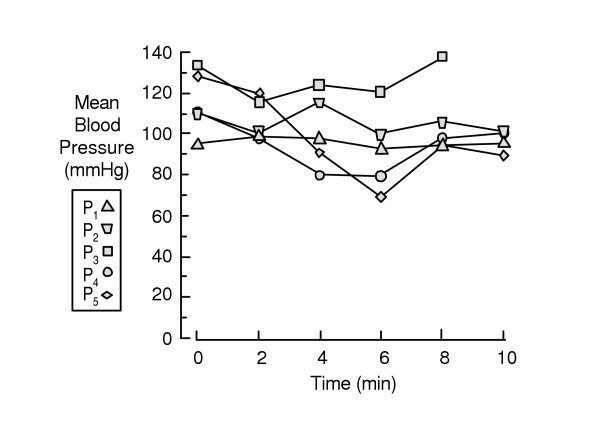
Mean blood pressure (MBP) was calculated from systolic and diastolicpressures for the 5 patients (P_1_–P_5_). MBP was measured before Ado infusion (time 0), and then every 2 min for the next 10 min, except in P_3 _in which a 10 min MBP was not obtained. Ado infusion caused MBP to decrease at some point during the infusion in all subjects, but the pressure did not decrease below 69 mmHg in any of the subjects.

The infusion of Ado caused MBP to decrease in most of the subjects (Figure 2), ranging from a 1 mmHg decrease in P_1 _(control MBP, 94 mmHg) to a 59 mmHg decrease in P_5 _(control MBP, 128 mmHg). This maximum decrease in MBP occurred after 2 minutes of Ado infusion in two subjects (P_2_, P_3_) and after 6 min of infusion in three subjects (P_1_, P_4_, P_5_). Note on Figure 2 that MBP did not decrease below 69 mmHg in any of the subjects.

The decrease in MBP was associated with decreases in both systolic (SBP) and diastolic (DBP) pressures (Table 1). Note also on Table 1 that adenosine increased heart rate (HR) significantly during the period of infusion. This increase in HR is typically seen in human subjects infused with adenosine and is thought to be a reflex response to the fall in blood pressure.

## Discussion

The results have shown that adenosine can increase plasma levels of VEGF protein in humans. The intravenous infusion of adenosine caused a three-fold increase in plasma VEGF protein levels one hour after the infusion (Figure 1a). Previous studies have shown that exogenous administration of adenosine or adenosine agonists (Hashimoto *et al. *1994; Fischer *et al. *1995; Takagi *et al. *1996; Pueyo *et al. *1998; Grant *et al. *1999; Gu *et al. *1999; Grant *et al. *2001; Leibovich *et al. *2002; Gardener & Olah, 2003; Feoktistov *et al. *2003) or upregulation of endogenous adenosine (Gu *et al. *2000) can enhance VEGF protein and/or mRNA expression in a variety of cultured cells (for review, see Adair, 2005). However, the present study is the first, to our knowledge, that demonstrates that the infusion of adenosine can increase circulating levels of VEGF *in vivo*.

The known cardiovascular effects of adenosine are mediated by the A_1 _and A_2 _(subtypes A_2A _and A_2B_) receptors (for review see Shryock & Belardinelli, 1997; Ralevic & Burnstock 1998; Rongen *et al. *1999). Responses to activation of the A_1 _receptor include slowing of the heart and slowing of impulse conduction through the atrioventricular node, reduction of atrial contractility, inhibition of beta-adrenergic effects, and vasodilatation (Bryan & Marshall, 1999). Activation of A_2 _receptors causes (a) vascular smooth muscle relaxation (Belardinelli *et al. *1998; Ngai *et al. *2001; Hinschen *et al. *2003), which can lead to decreases in blood pressure, and (b) induction of VEGF expression from a variety of cell types (Hashimoto *et al. *1994; Pueyo *et al. *1998; Grant *et al. *1999; Gu *et al. *1999; Leibovich *et al. *2002; Gardener & Olah, 2003; Feoktistov *et al. *2003). Recent development of selective agonists and antagonists for A_2 _receptor subtypes suggests that vasodilatation is mediated by A_2A _(Shryock & Belardinelli, 1997; Belardinelli *et al. *1998; Rongen *et al. *1999) and perhaps A_2B _receptors (Ralevic & Burnstock 1998; Ngai *et al. *2001; Hinschen *et al. *2003). The A_2B _receptor subtype appears to mediate the induction of VEGF protein and mRNA in various human cell types. Grant *et al*. (1999) have shown that activation of the adenosine A_2B _receptor (but not the A_2A _receptor) can increase VEGF mRNA and protein expression in cultured human retinal endothelial cells. Feoktistov *et al*. (2003) established that adenosine acting by way of the A_2B _receptor can stimulate VEGF expression in human mast cells. These latter investigators (Feoktistov *et al*. 2002) also showed that adenosine can induce VEGF expression in human microvascular endothelial cells, where A_2B _receptors are abundant, but not in human umbilical vein endothelial cells, which preferentially express A_2A _receptors. Therefore, the available data suggest that adenosine induced vasodilatation and VEGF expression could be mediated to some extent by similar receptors.

It is not clear from the present study whether the infusion of adenosine actually induced the transcription and translation of VEGF ; however, it is known that both VEGF mRNA and protein can be induced within a one hour period of time. Breen *et al*. (1996) found a fourfold increase in VEGF mRNA at the termination of a one hour bout of treadmill exercise in rats. Gu and Adair (1997) showed that exposing dog myocardial vascular smooth muscle cells to a hypoxic environment caused a twofold increase in VEGF protein concentration in the media after only one hour of exposure to the hypoxic environment. Therefore, it is conceivable that adenosine could induce VEGF transcription and translation resulting in elevated plasma levels of VEGF at one hour post infusion. However, additional studies will be required to determine whether the increase in circulating VEGF caused by adenosine did in fact result from adenosine-induced production of VEGF.

A possibility that must be considered is that the transient decrease in blood pressure caused by adenosine leads to transient hypoxia in the tissues, and that the hypoxia, in turn, leads to the induction of VEGF expression. Hypoxia is a well-known stimulus for the induction of VEGF expression both *in vitro *and *in vivo *(for review see Ferrara & Davis-Smyth, 1997; Ferrara, 2001). However, it seems unlikely that this indirect induction of VEGF by adenosine could explain entirely the results of the present study for the following reasons: (a) adenosine can induce VEGF mRNA and protein expression *in vitro *where blood pressure is not an issue, as discussed previously; (b) adenosine increased the plasma levels of VEGF in two patients in whom the blood pressure had decreased by less than 15 mmHg, a decrease that should not cause significant perfusion problems or hypoxia in the tissues; (c) mean blood pressure did not fall below 69 mmHg in any of the subjects; and (d) none of the subjects experienced symptoms suggestive of cerebral hypoxia during the infusion of adenosine. All subjects were supine during the infusion of adenosine to minimize potential perfusion problems. Furthermore, adenosine induced vasodilatation would be expected to improve the distribution of oxygen within a tissue, also arguing against tissue hypoxia. Therefore, it is unlikely that the adenosine induced decrease in blood pressure contributed significantly to the rise in VEGF.

## Conclusion

In conclusion, this study provides preliminary data showing that an intravenous infusion of adenosine can increase plasma levels of VEGF in humans. Additional studies will be required to determine whether the increase in plasma VEGF caused by adenosine results from the transcription and translation of VEGF.

## Methods

### Subject demographics

The study protocol was approved by the University of Mississippi Medical Center Internal Review Board. The subjects underwent routine sestamibi myocardial perfusion scans because of suspected coronary artery disease. They consisted of one man and four women; mean age was 49.3 ± 6.7 years and mean weight was 88.2 ± 8.5 kg. None of the subjects had taken caffeine prior to the test, had a history of asthma or myocardial infarction, or were being treated with blood pressure medications. Their mean blood pressure (MBP, auscultatory sphygmomanometry) prior to testing was 116 ± 7 mmHg (systolic, 159 ± 12 mmHg; diastolic, 94 ± 5 mmHg). MBP was calculated as the diastolic pressure plus one third of the pulse pressure. Mean heart rate was 70 ± 3 beats per minute; one patient (P_4_) was paced at 66 beats per minute. Also, one individual (P_3_) had a history of multiple myeloma and was being evaluated for kidney transplantation.

### Experimental protocol

Adenosine (Adenoscan^®^, Medco Research, Inc.) was infused intravenously at 0.14 mg kg^-1 ^min^-1 ^for six minutes (i.e., total dose was 0.84 mg kg^-1^). MBP was measured before the adenosine infusion and then every two minutes for the next ten minutes. Technetium-99 (sestamibi) was administered three minutes into the adenosine infusion and a nuclear scan was performed. The infusion of Ado was well tolerated by all subjects. There were no significant ECG abnormalities during the test and no myocardial perfusion abnormalities were found. None of the subjects were diagnosed with cardiovascular disease. Three mL of blood were collected in EDTA coated tubes before the adenosine infusion and at 1, 2, and 8 h after the adenosine infusion. The blood samples were centrifuged at 700 g for 5 minutes at 4°C, and plasma was stored at -80°C.

### Measurement of VEGF protein

Plasma levels of VEGF were measured in duplicate by sandwich ELISA (R&D Systems, Minneapolis). In these assays, the test sample is sandwiched between a monoclonal antibody against human recombinant VEGF. A second polyclonal antibody against VEGF is conjugated to horseradish peroxidase and then added to the mixture. Color develops by addition of hydrogen peroxide and chromagen tetramethylbenzidine, and the intensity is measured at 450 nm. VEGF plasma protein levels (pg ml^-1^) were calculated using an equation generated from the regression analysis of standard VEGF concentrations.

### Statistical analyses

The results are expressed as means ± standard errors of the mean (S.E.M.). Differences between the groups (e.g., plasma VEGF at various times) were compared using analysis of variance for repeated measurements and Dunnett's multiple comparison test (InStat, GraphPad Software, Inc.).

## Abbreviations

Ado, adenosine

VEGF, vascular endothelial growth factor

HR, heart rate

MBP, mean blood pressure

SBP, systolic blood pressure

DBP, diastolic blood pressure

## Authors' contributions

Thomas H. Adair, Jean-Pierre Montani, and Reid Cotten designed the study. Reid Cotten, Kenneth R. Bennett, and Michael R. McMullan performed the myocardial perfusion scans and collected the blood samples. Thomas H. Adair, Jian-Wei Gu, Janelle S. Pryor, and Preston B. McDonnell performed the various analyses. All authors contributed to writing the manuscript.

## Figures and Tables

**Table 1:** Acute cardiovascular effects of adenosine infusion.

	0 min	2 min	4 min	6 min	8 min	10 min
HR	70 ± 3	77 ± 4	81 ± 5*	82 ± 5*	78 ± 4	77 ± 5
SBP	159 ± 12	153 ± 9	144 ± 11	135 ± 9*	155 ± 12	145 ± 7
DBP	94 ± 5	80 ± 5	72 ± 8*	68 ± 9**	81 ± 7	72 ± 6
MBP	116 ± 7	104 ± 6	96 ± 8*	90 ± 9**	106 ± 8	96 ± 3

Heart rate (HR, beats per minute), systolic blood pressure (SBP, mmHg), diastolic blood pressure (DBP, mmHg) and mean blood pressure (MBP, mmHg) were measured before Ado infusion (0 min) and then every 2 min for the next 10 min. MBP was calculated from SBP and DBP. Values are means ± S.E.M. (n = 5 at all times, except at 10 min where n = 4). * indicate p < 0.05. ** indicate p < 0.01.

## References

[B1] Adair TH (2005). Growth regulation of the vascular system: an emerging role for adenosine. Am J Physiol Regal Integr Comp Physiol.

[B2] Adair TH, Gay WJ, Hester RL, Montani JP, Imai S, Nakazawa M (1991). Does adenosine have a regulatory role in the growth of blood vessels?. Role of adenosine and adenine nucleotides in the biological system.

[B3] Adair TH, Gay WJ, Montani JP (1990). Growth regulation of the vascular system: evidence for a metabolic hypothesis. Am J Physiol.

[B4] Adair TH, Montani JP, Strick DM, Guyton AC (1989). Vascular development in chick embryos: a possible role for adenosine. Am J Physiol.

[B5] Belardinelli L, Shryock JC, Snowdy S, Zhang Y, Monopoli A, Lozza G, Ongini E, Olsson RA, Dennis DM (1998). The A_2a _adenosine receptor mediates coronary vasodilation. J Pharmacol Exp Ther.

[B6] Berne RM (1980). The role of adenosine in the regulation of coronary blood flow. Circ Res.

[B7] Breen EC, Johnson EC, Wagner H, Tseng HM, Sung LA, Wagner PD (1996). Angiogenic growth factor mRNA responses in muscle to a single bout of exercise. J Appl Physiol.

[B8] Bryan PT, Marshall JM (1999). Adenosine receptor subtypes and vasodilatation in rat skeletal muscle during systemic hypoxia: a role for A_1 _receptors. J Physiol.

[B9] Deussen A, Borst M, Kroll K, Schrader J (1988). Formation of S-adenosylhomocysteine in the heart. II: a sensitive index for regional myocardial underperfusion. Circ Res.

[B10] Dobson JG, Ordway RW, Fenton RA (1986). Endogenous adenosine inhibits catecholamine contractile responses in normoxic hearts. Am J Physiol.

[B11] Ely SW, Berne RM (1992). Protective effects of adenosine in myocardial ischemia. Circulation.

[B12] Ethier MF, Chander V, Dobson JG (1993). Adenosine stimulates proliferation human endothelial cells in culture. Am J Physiol.

[B13] Ethier MF, Dobson JG (1997). Adenosine stimulation of DNA synthesis in human endothelial cells. Am J Physiol.

[B14] Feoktistov I, Goldstein AE, Ryzhov S, Zeng D, Belardinelli L, Voyno-Yasenetskaya T, Biaggioni I (2002). Differential expression of adenosine receptors in human endothelial cells: role of A_2B _receptors in angiogenic factor regulation. Circ Res.

[B15] Feoktistov I, Ryzhov S, Goldstein AE, Biaggioni I (2003). Mast cell-mediated stimulation of angiogenesis: cooperative interaction between A_2B _and A_3 _adenosine receptors. Circ Res.

[B16] Ferrara N (2001). Role of vascular endothelial growth factor in regulation of physiological angiogenesis. Am J Physiol.

[B17] Ferrara N, Davis-Smyth T (1997). The biology of vascular endothelial growth factor. EndocrRev.

[B18] Fischer S, Sharma HS, Karliczek GF, Schaper W (1995). Expression of vascular permeability factor / vascular endothelial growth factor in pig cerebral microvascular endothelial cells and its upregulation by adenosine. Brain Res Mol Brain Res.

[B19] Gardner AM, Olah ME (2003). Distinct protein kinase C isoforms mediate regulation of vascular endothelial growth factor expression by A_2A _adenosine receptor activation and phorbol esters in pheochromocytoma PC12 cells. J Biol Chem.

[B20] Grant MB, Davis MI, Caballero S, Feoktistov I, Biaggioni I, Belardinelli L (2001). Proliferation, migration, and ERK activation in human retinal endothelial cells through A_2B _adenosine receptor stimulation. Invest Ophthalmol Vis Sci.

[B21] Grant MB, Tarnuzzer RW, Caballero S, Ozeck MJ, Davis MI, Spoerri PE, Feoktistov I, Biaggioni I, Shryock JC, Belardinelli L (1999). Adenosine receptor activation induces vascular endothelial growth factor in human retinal endothelial cells. Circ Res.

[B22] Gu JW, Adair TH (1997). Hypoxia-induced expression of VEGF is reversible in myocardial vascular smooth muscle cells. Am J Physiol.

[B23] Gu JW, Brady AL, Anand V, Moore MC, Kelly WC, Adair TH (1999). Adenosine upregulates VEGF Expression in cultured myocardial vascular smooth muscle cells. Am J Physiol.

[B24] Gu JW, Ito BR, Sartin A, Frascogna N, Moore M, Adair TH (2000). Inhibition of adenosine kinase induces expression of VEGF mRNA and protein in myocardial myoblasts. Am J Physiol.

[B25] Hashimoto E, Kage K, Ogita T, Nakaoka T, Matsuoka R, Kira Y (1994). Adenosine as an endogenous mediator of hypoxia for induction of vascular endothelial growth factor mRNA in U-937 cells. Biochem Biophys Res Commun.

[B26] Hinschen AK, Rose'Meyer RB, Headrick JP (2003). Adenosine receptor subtypes mediating coronary vasodilation in rat hearts. J Cardiovasc Pharmacol.

[B27] Leibovich SJ, Chen JF, Pinhal-Enfield G, Belem PC, Elson G, Rosania A, Ramanathan M, Montesinos C, Jacobson M, Schwarzschild MA, Fink JS, Cronstein B (2002). Synergistic up-regulation of vascular endothelial growth factor expression in murine macrophages by adenosine A_2A _receptor agonists and endotoxin. Am J Pathol.

[B28] Marshall JM (2001). Roles of adenosine and nitric oxide in skeletal muscle in acute and chronic hypoxia. Adv Exp Med Biol.

[B29] Meininger CJ, Granger HJ (1990). Mechanisms leading to adenosine-stimulated proliferation of microvascular endothelial cells. Am J Physiol.

[B30] Meininger CJ, Schelling ME, Granger HJ (1988). Adenosine and hypoxia stimulate proliferation and migration of endothelial cells. Am J Physiol.

[B31] Neumann J, Gupta RC, Bodor GS, Bartel S, Kruse EG, Pask HT, Schmitz W, Scholz H, Watanabe AM (1995). Interaction of beta-adrenoceptor and adenosine receptor agonists on phosphorylation: identification of target proteins in mammalian ventricles. J Mol Cell Cardiol.

[B32] Ngai AC, Coyne EF, Meno JR, West GA, Winn HR (2001). Receptor subtypes mediating adenosine-induced dilation of cerebral arterioles. Am J Physiol.

[B33] Pueyo ME, Chen Y, D'Angelo G, Michel JB (1998). Regulation of vascular endothelial growth factor expression by cAMP in rat aortic smooth muscle cells. Exp Cell Res.

[B34] Ralevic V, Burnstock G (1998). Receptors for purines and pyrimidines. Pharmacol Rev.

[B35] Richardson RS, Wagner H, Mudaliar SR, Henry R, Noyszewski EA, Wagner PD (1999). Human VEGF gene expression in skeletal muscle: effect of acute normoxic and hypoxic exercise. Am J Physiol.

[B36] Rongen GA, Brooks SC, Pollard MJ, Ando S, Dajani HR, Notarius CF, Floras JS (1999). Effect of adenosine on heart rate variability in humans. Clin Sci (Lond).

[B37] Shryock JC, Belardinelli L (1997). Adenosine and adenosine receptors in the cardiovascular system: biochemistry, physiology, and pharmacology. Am J Cardiol.

[B38] Schrader J (1990). Adenosine: a homeostatic metabolite in cardiac energy metabolism. Circulation.

[B39] Sparks HV, Bardenheuer H (1986). Regulation of adenosine formation by the heart. Circ Res.

[B40] Stepp DW, Van Bibber R, Kroll K, Feigl EO (1996). Quantitative relation between interstitial adenosine concentration and coronary blood flow. Circ Res.

[B41] Takagi H, King GL, Robinson GS, Ferrara N, Aiello LP (1996). Adenosine mediates hypoxic induction of vascular endothelial growth factor in retinal pericytes and endothelial cells. Invest Ophthalmol Vis Sci.

[B42] Teuscher E, Weidlich W (1985). Adenosine nucleotides, adenosine and adenine as angiogenesis factors. Biomed Biochim Acta.

